# Supplementary material for: The Mechanisms of GPR55 Receptor Functional Selectivity during Apoptosis and Proliferation Regulation in Cancer Cells
Source: Int J Mol Sci. 2023 Mar 14;24(6):5524. doi: 10.3390/ijms24065524 (PMC10054013; doi:10.3390/ijms24065524)
Supplement: Supplementary file 1 [file ijms-24-05524-s001.zip › ijms-2164904-supplementary.pdf]

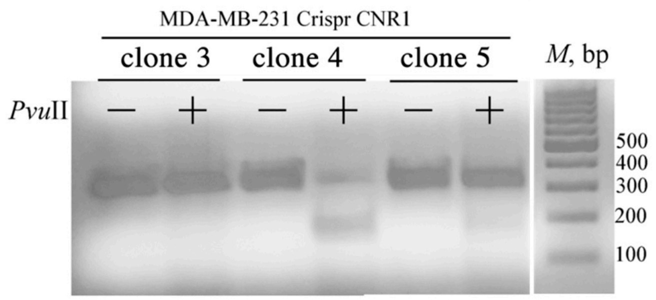

Figure S1. The evaluation of the CB1 receptor knockout efficiency. Electrophoresis of the *CB1* (CNR1) genomic DNA fragments obtained by PCR before (-) and after (+) PvuII restriction enzyme treatment; the disappearance of the PvuII cleavage would indicate the loss of the appropriate part of the gene, equaling to the successful gene modification.

```
CNR1   CAACAAGTCTCTCTCGTCCTTCAAGGAGAATGAGGAGAACATCCAGTGTGGGGAGAACTT 124
3.3    CAACAAGTCTCTCTCGTCCTTCAAGGAGAATGAGGAGAACATCCAGTGTGGGGAGAACTT 238
3.2    CAACAAGTCTCTCTCGTCCTTCAAGGAGAATGAGGAGAACATCCAGTGTGGGGAGAACTT 238
3.4    CAACAAGTCTCTCTCGTCCTTCAAGGAGAATGAGGAGAACATCCAGTGTGGGGAGAACTT 236
3.5    CAACAAGTCTCTCTCGTCCTTCAAGGAGAATGAGGAGAACATCCAGTGTGGGGAGAACTT 235
3.1    CAACAAGTCTCTCTCGTCCTTCAAGGAGAATGAGGAGAACATCCAGTGTGGGGAGAACTT 238
3.7    CAACAAGTCTCTCTCGTCCTTCAAGGAGAATGAGGAGAACATCCAGTGTGGGGAGAACTT 236
       ************************************************************

CNR1   CATGGACATAGAGTGTTTCATGGTCCTGAACCCCAGCCAGC-AGCTGGCCATTGCAGTCC 183
3.3    CGTGGACATAGAGTGTTTCATGGTCCT--------------------------------- 265
3.2    CATGGACATAGAGTGTTTCATGGTCCTGAACCCCAGCCAGCAAGCTGGCCATTGCAGTCC 298
3.4    CATGGACATAGAGTGTTTCATGGTCCTGAACCCCA-------AGCTGGCCATTGCAGTCC 289
3.5    CATGGACATAGAGTGTTTCATGGTCCTGAACCCCAGCCA---AGCTGGCCATTGCAGTCC 292
3.1    CATGGACATAGAGTGTTTCATGGTCCTGAACCCCAGCCA---AGCTGGCCATTGCAGTCC 295
3.7    CATGGACATAGAGTGTTTCATGGTCCTGAACCCCAGCCA---AGCAGGCCATTGCAGTCC 293
       * *************************

CNR1   TGTCCCTCACGCTGGGCACCTTCACGGTCCTGGAGAACCTCCTGGTGCTGTGCGTCATCC 243
3.3    -GTCCCTCACGCTGGGCACCTTCACGGTCCTGGAGAACCTCCTGGTGCTGTGCGTCATCC 324
3.2    TGTCCCTCACGCTGGGCACCTTCACGGTCCTGGAGAACCTCCTGGTGCTGTGCGTCATCC 358
3.4    TGTCCCTCACGCTGGGCACCTTCACGGTCCTGGAGAACCTCCTGGTGCTGTGCGTCATCC 349
3.5    TGTCCCTCACGCTGGGCACCTTCACGGTCCTGGAGAACCTCCTGGTGCTGTGCGTCATCC 352
3.1    TGTCCCTCACGCTGGGCACCTTCACGGTCCTGGAGAACCTCCTGGTGCTGTGCGTCATCC 355
3.7    TGTCCCTCACGCTGGGCACCTTCACGGTCCTGGAGAACCTCCTGGTGCTGTGCGTCATCC 353
        ***********************************************************
```

Figure S2. The evaluation of the CB1 receptor knockout efficiency. Sequencing results alignment with the native *CNR1* mRNA. *Pvu*II cleavage site is shown in bold.

```
cnr2  TCCTGCTGAAGATTGGCAGCGTGACTATGACCTTCACAGCCTCTGTGGGTAGCCTCCTGC  65
2e5   TCCTGCTGAAGATTGGCAGCGTGACTATGACCTTCACAGCCTCTGTGGGTAGCCTCCTGC  179
1h9   TCCT-CTGAAGATTGGCAGCGTGACTATGACCTTCACAGCCTCTGTGGGTAGCCTCCTGC  176
1h6   TCCTGCTGAAGATTGGCAGCGTGACTATGACCTTCACAGCCTCTGTGGGTAGCCTCCTGC  176
2f1   TCCTGCTGAAGATTGGCAGCGTGACTATGACCTTCACAGCCTCTGTGGGTAGCCTCCTGC  178
1b10  TCCTGCTGAAGATTGGCAGCGTGACTATGACCTTCACAGCCTCTGTGGGTAGCCTCCTGC  179
1b3   TCCTGCTGAAGATTGGCAGCGTGACTATGACCTTCACAGCCTCTGTGGGTAGCCTCCTGC  179
      **** *******************************************************

cnr2  TGACCGCCATTGACCGATACCTCTGCCTGCGCTATCCACCTTCCTACAAAGCTCTGCTCA  125
2e5   TGACCGCCATTGACCGATACCTCTGCCTGCGCTATCCACCTTCCTACAAAGCTCTGCTCA  239
1h9   TGACCGCCATTGACCGATACCTCTGCCTGCGCTATCCACCTTCCTACAAAGCTCTGCTCA  236
1h6   TGACCGCCATTGACCGATACCTCTGCCTGCGCTATCCACCTTCCTACAAAGCTCTGCTCA  236
2f1   TGACCGCCATTGACCGATACCTCTGCCTGCGCTATCCACCTTCCTACAAAGCTCTGCTCA  238
1b10  TGACCGCCATTGACCGATACCTCTGCCTGCGCTATCCACCTTCCTACAAAGCTCTGCTCA  239
1b3   TGACCGCCATTGACCGATACCTCTGCCTGCGCTATCCACCTTCCTACAAAGCTCTGCTCA  239
      ************************************************************

cnr2  CCCGTGGAAGGGCACTGGTGACCCTGGGCATCATGTGGGTCCTCTCAGCACTAGTCTCCT  185
2e5   CCCGTGGAAGGGCACTGGTGAC-CTGGGCATCATGTGGGTCCTCTCAGCACTAGTCTCCT  298
1h9   CCCGTGGAAGGGCACTGG---CCCTGGGTATCATGTGGGTCCTCTCAGCACTAGTCTCCT  293
1h6   CCCGTGGAAGGGCACTGGTGAC-CTGGGCATCATGTGGGTCCTCTCAGCACTAGTCTCCT  295
2f1   CCCGTGGAAGGGCACTGGTGA--CTGGGCATCATGTGGGCCCTCTCAGCACTAGTCTCCT  297
1b10  CCCGTGGAAGGGCACTGGTGAC-CTGGGCATCATGTGGGTCCTCTCAGCACTAGTCTCCT  298
1b3   CCCGTGGAAGGGCACTGGTGAC-CTGGGCATCATGTGGGTCCTCTCAGCACTAGTCTCCT  298
      ******************   * ***** ********** ********************
```

Figure S3A. The evaluation of the CB2 receptor knockout efficiency. Sequencing results alignment with the native *CNR2* mRNA. PasI cleavage site is shown in bold.

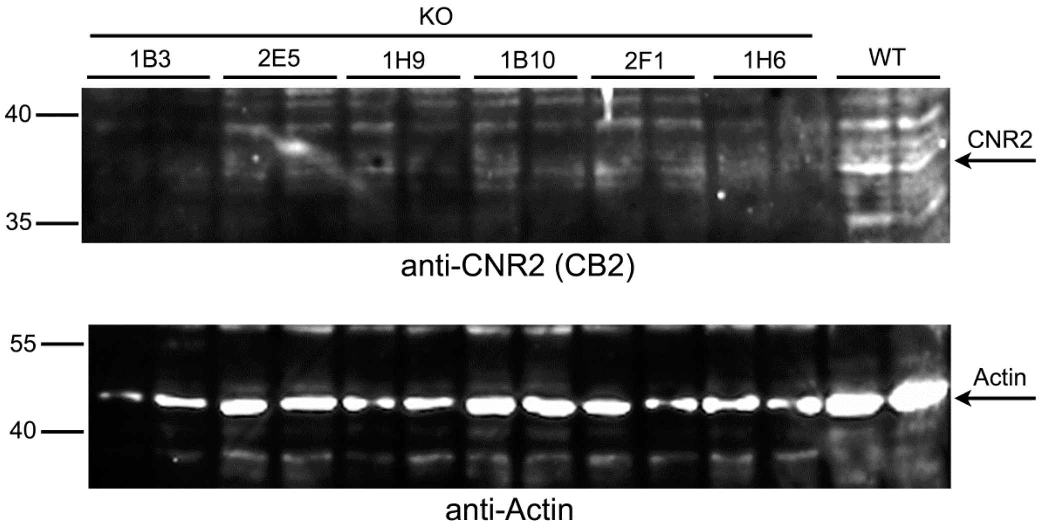

Figure S3B. The evaluation of the CB2 receptor knockout efficiency. Western blot data.

GPR55   GCATCCCTATCTACAGTTT**CCATGG**GAAAGTGGAAAAATACATGTGCTTCCACAACATGT 231
1B4   GCATCCCTATCTACAGTTTC---GGGAAAGTGGAAAAATACATGTGCTTCCACAACATGT 295
C12   GCATCCCTATCTACAGTTTC—-TGGGAAAGTGGAAAAATACATGTGCTTCCACAACATGT 298
F10   GCATCCCTATCTA-------–-------AGTGGAAAAATACATGTGCTTCCACAACATGT 296
        *************   ********************************

Figure S4A. The evaluation of the GPR55 receptor knockout efficiency. Sequencing results alignment with the native *GPR55* mRNA. NcoI cleavage site is shown in bold.

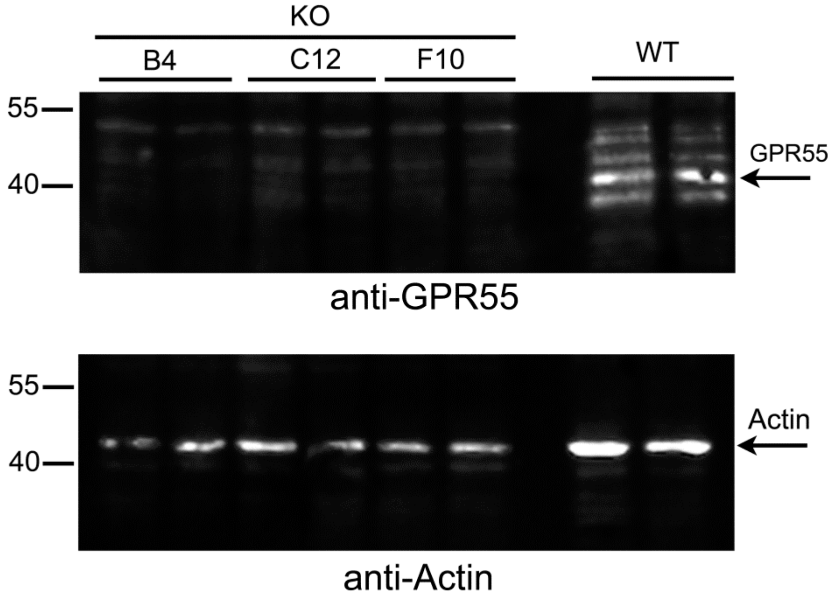

Figure S4B. The evaluation of the GPR55 receptor knockout efficiency. Western blot data.

GPR18   CATTGCTTTATGGCTTCTTGCCTTTATTAGTGCTGACAGATACATGGCCATT**TGTACA**GCC 189
1f2   CATTGCTTTATGGCTTCTTGCCTTTATTAGTGCTGACAGATACATGGCCATT-TACTGCC 299
2d9   CATTGCTTTATGGCTTCTTGCCTTTGTTAGTGCTGACAGATACATGGCCATT-TACAGCC 299
1h7   CATTGCTTTATGGCTTCTTGCCTTTATTAGTGCTGACAGATACATGGCCATT-TACAGCC 298
2b9   CATTGCTTTATGGCTTCTTGCCTTTATTAGTGCTGACAGATACATGGCCATT-TACAGCC 298
        ************************* ************************** *** ***

Figure S5. The evaluation of the GPR18 receptor knockout efficiency. Sequencing results alignment with the native *GPR18* mRNA. BstAUI cleavage site is shown in bold.
